# Supplementary figures and images for: Larazotide acetate induces recovery of ischemia-injured porcine jejunum via repair of tight junctions
Source: PLoS One. 2021 Apr 22;16(4):e0250165. doi: 10.1371/journal.pone.0250165 (PMC8061941; doi:10.1371/journal.pone.0250165)

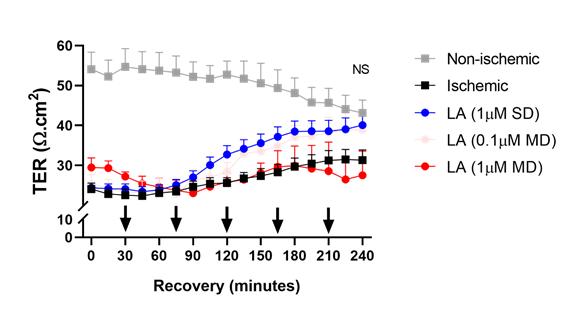

Supplement: S1 Fig — LA was applied to the apical bathing solution after the 30-minute equilibration period and every 45-minutes following the first dose. No statistical significance was detected between specific timepoint comparisons. Values are means ± SEM; n ≥ 3. (TIF) [file pone.0250165.s001.TIF]
