# Supplementary material for: Larazotide acetate induces recovery of ischemia-injured porcine jejunum via repair of tight junctions
Source: PLoS One. 2021 Apr 22;16(4):e0250165. doi: 10.1371/journal.pone.0250165 (PMC8061941; doi:10.1371/journal.pone.0250165)
Supplement: S1 Raw images — (PDF) [file pone.0250165.s002.pdf]

Cytoplasmic fractions: claudin-4

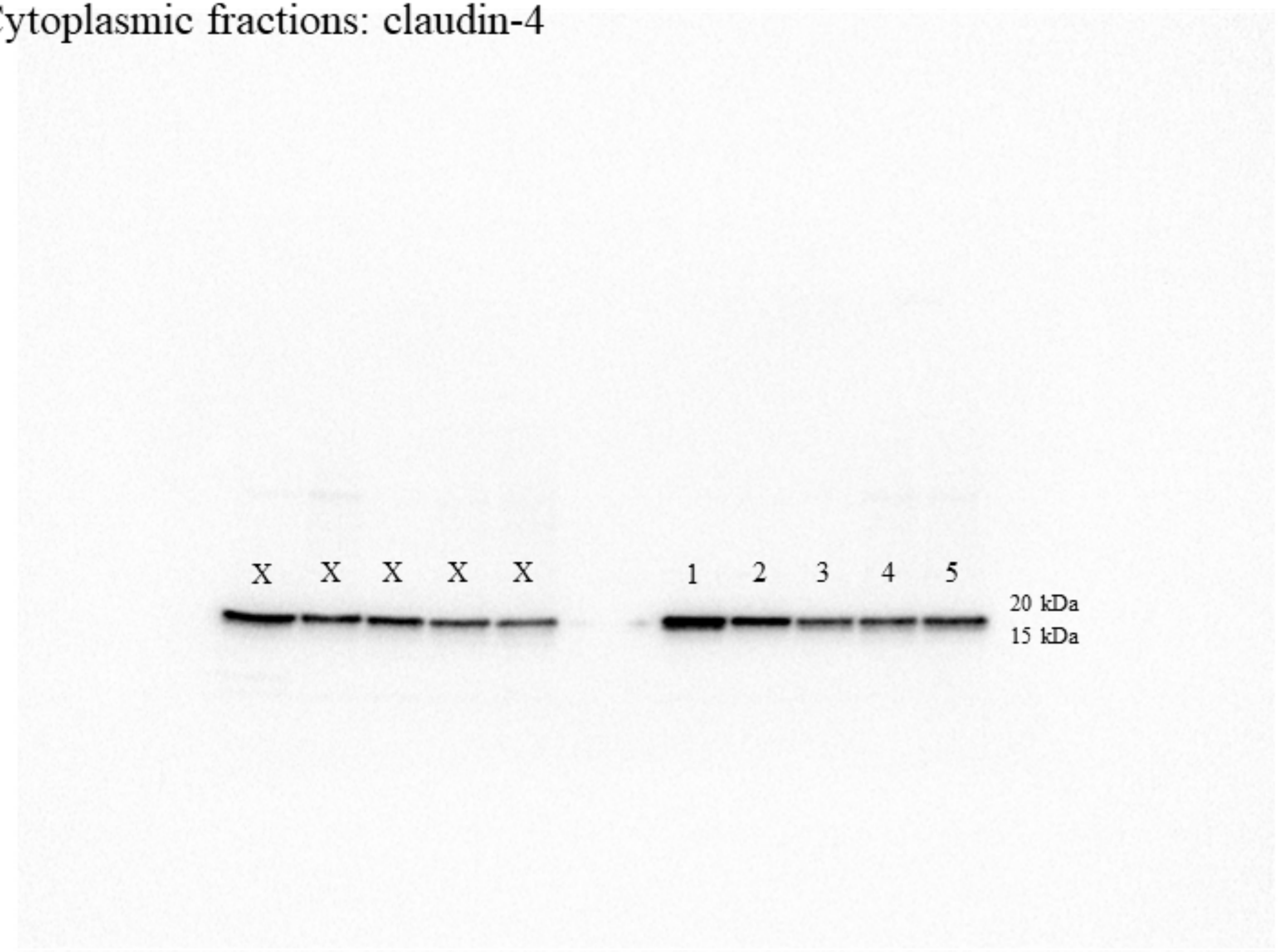

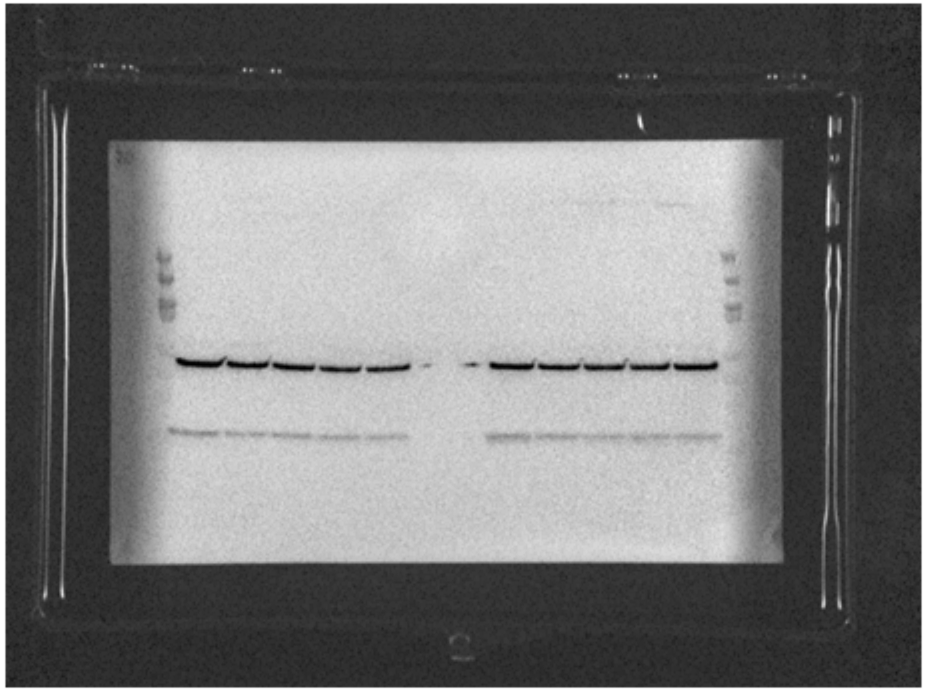

Cytoplasmic fractions: β-actin

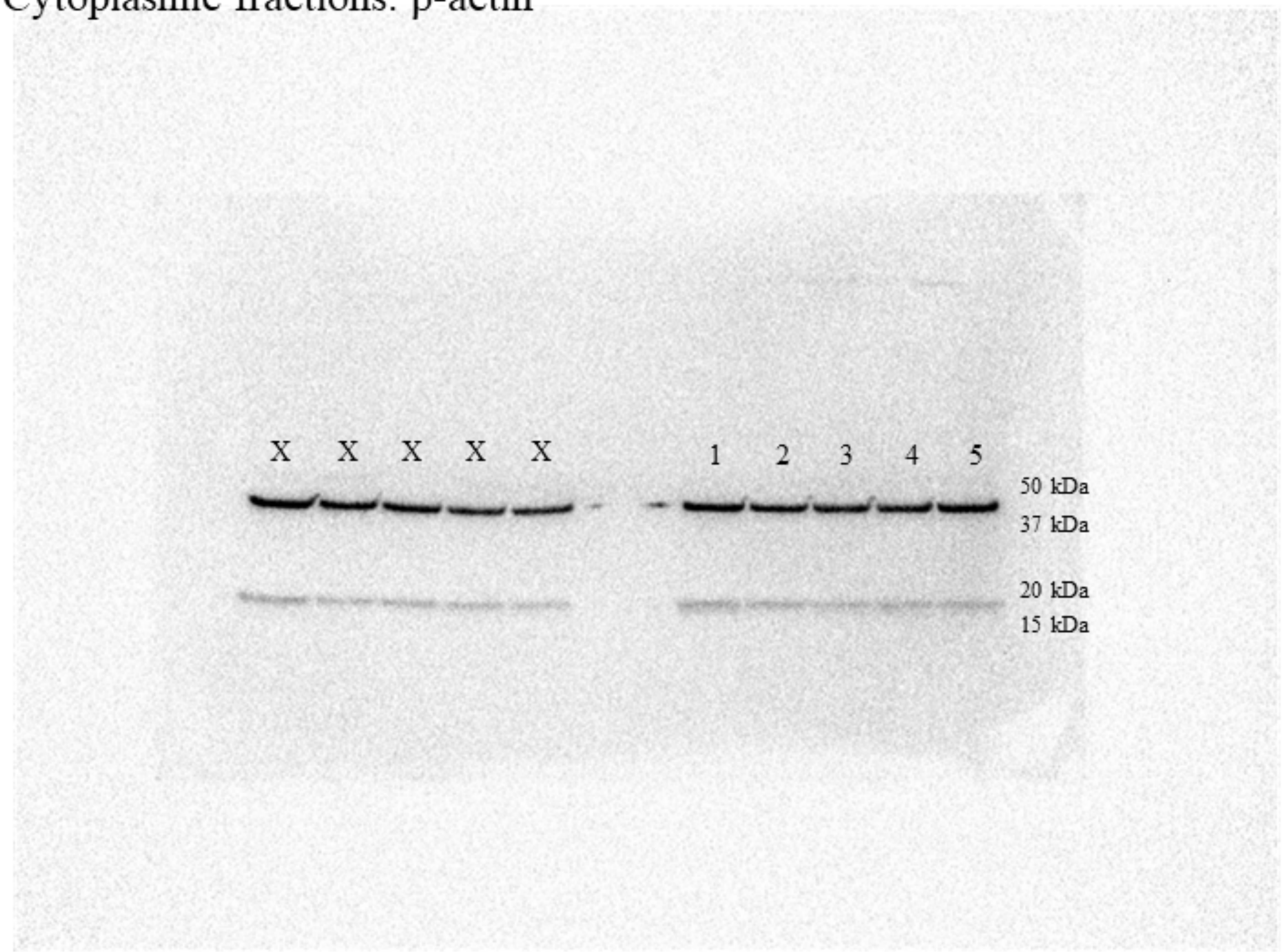

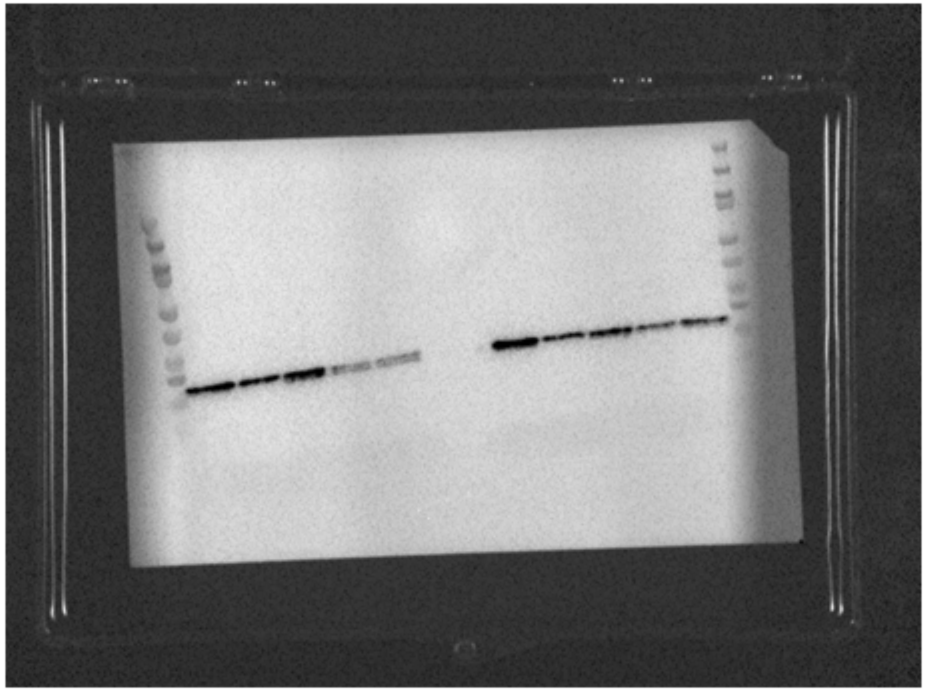

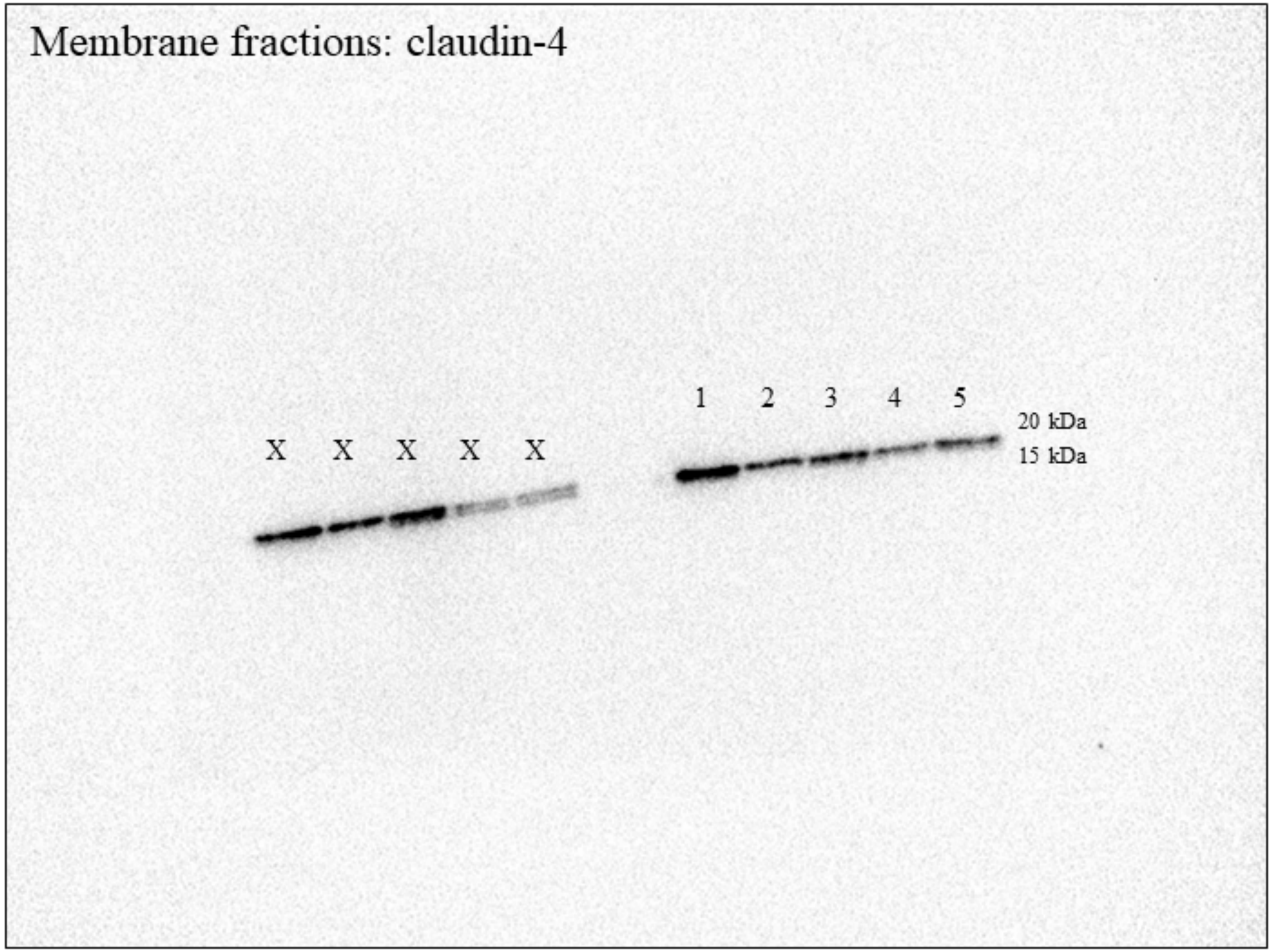
Membrane fractions: claudin-4
X X X X X
1 2 3 4 5
20 kDa
15 kDa

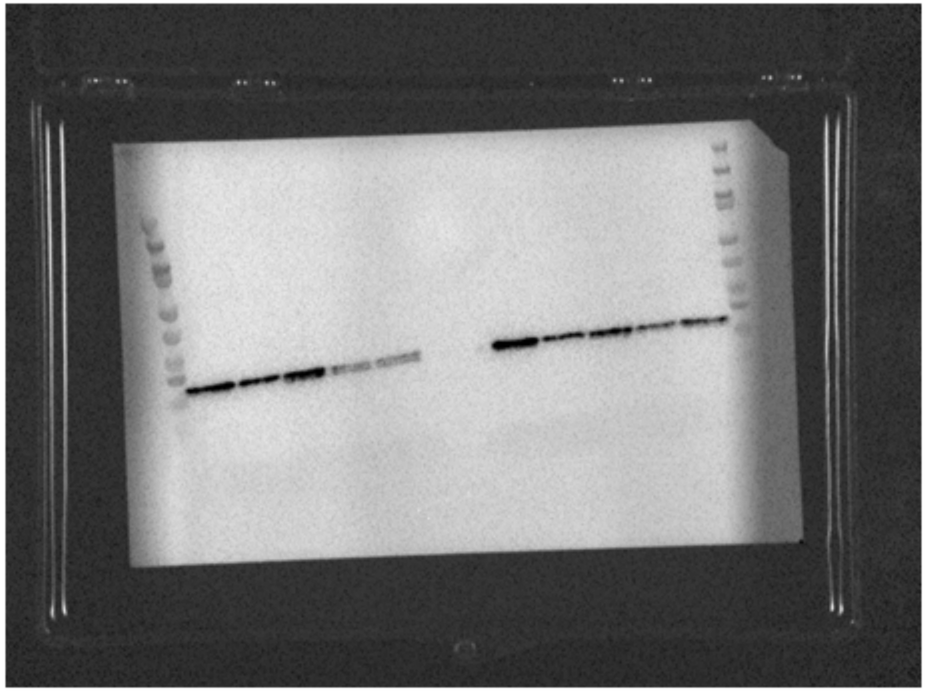

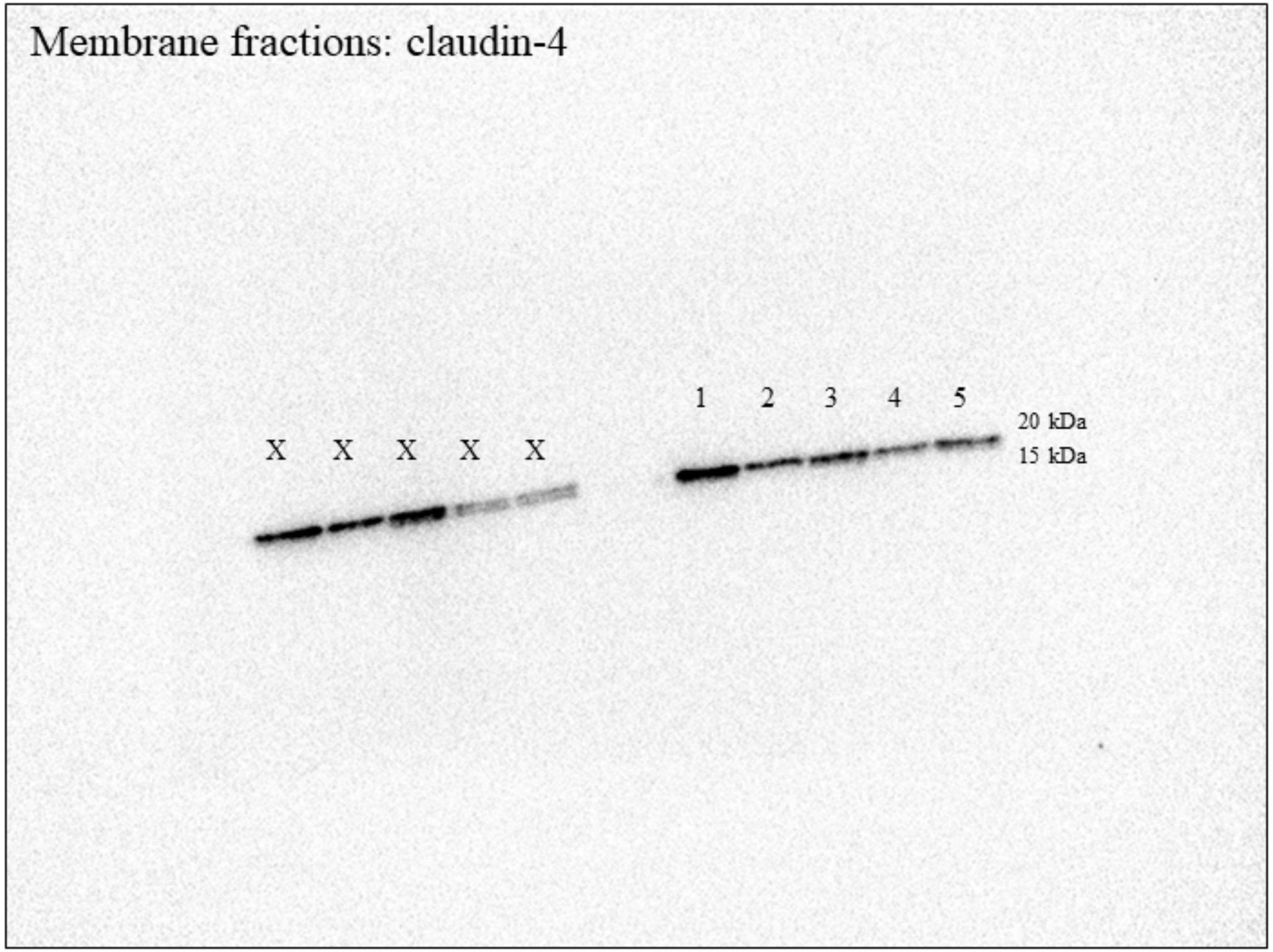
Membrane fractions: claudin-4
X X X X X
1 2 3 4 5
20 kDa
15 kDa

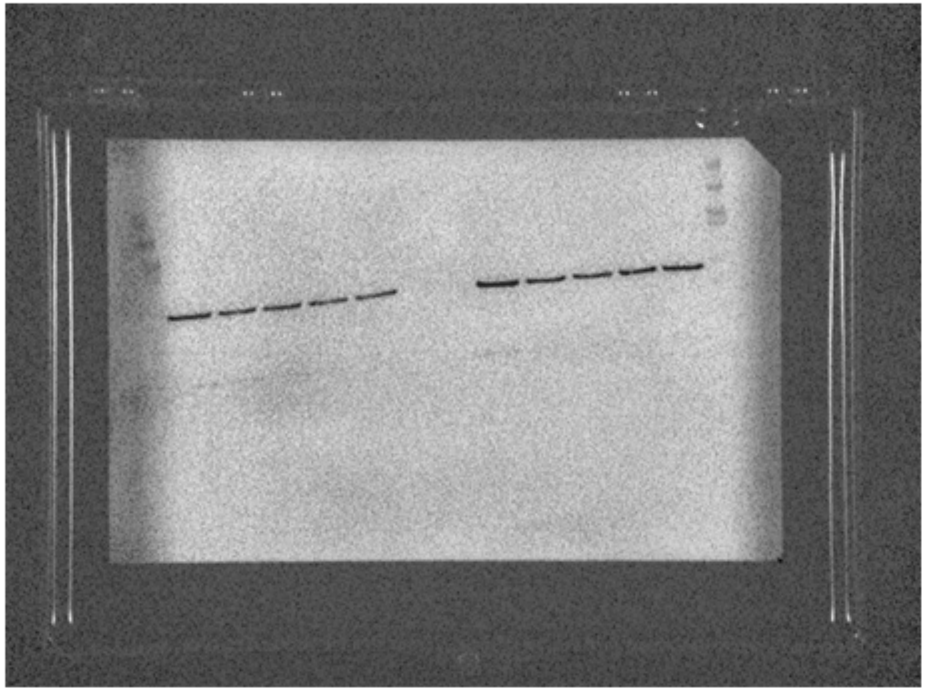

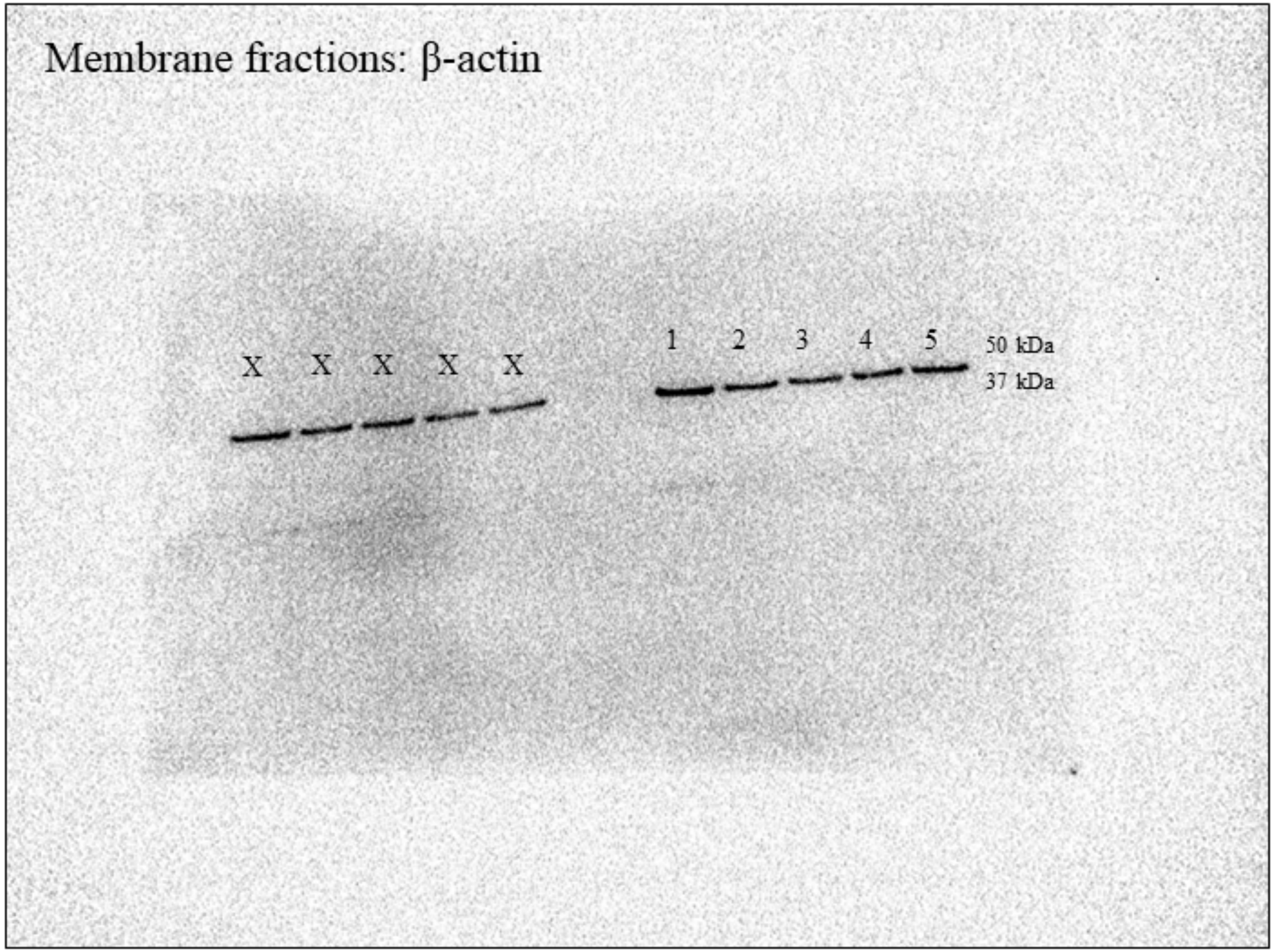
Membrane fractions: β-actin
X
X
X
X
X
1
2
3
4
5
50 kDa
37 kDa

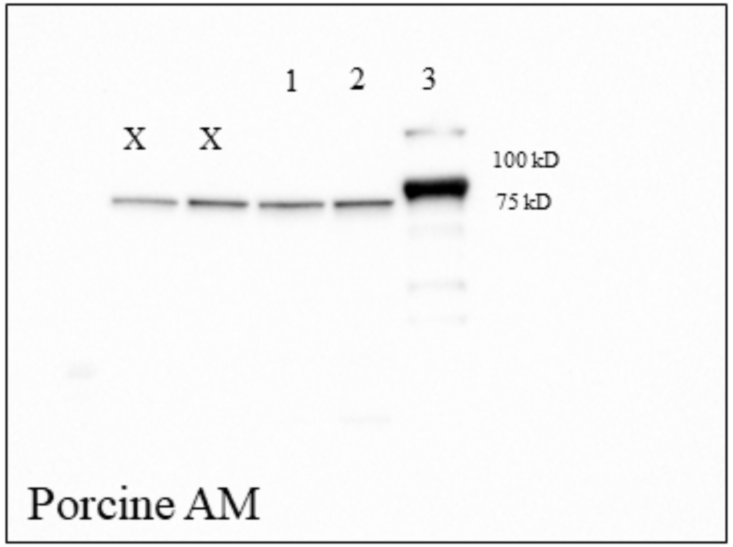
1
2
3
X
X
100 kD
75 kD
Porcine AM

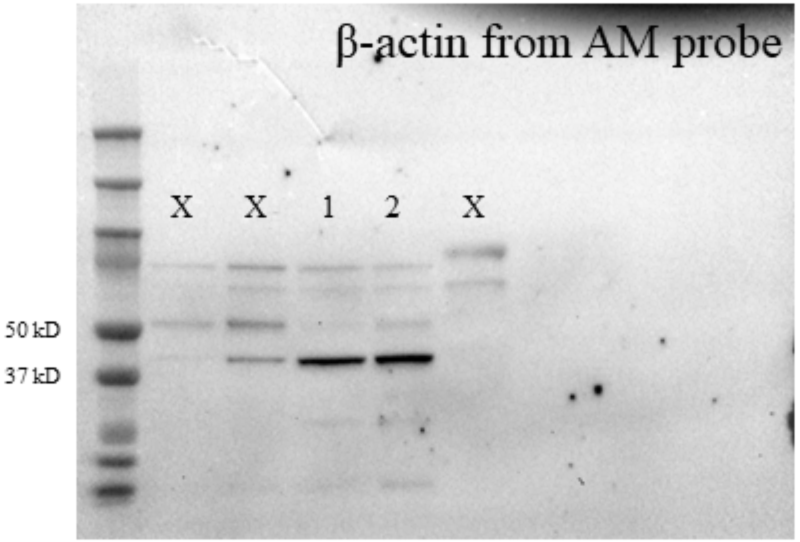
β-actin from AM probe
X
X
1
2
X
50kD
37kD
