# Supplementary material for: Larazotide acetate induces recovery of ischemia-injured porcine jejunum via repair of tight junctions
Source: PLoS One. 2021 Apr 22;16(4):e0250165. doi: 10.1371/journal.pone.0250165 (PMC8061941; doi:10.1371/journal.pone.0250165)
Supplement: Attachment — Submitted filename: Response to Reviewers.docx [file pone.0250165.s003.docx]

Reviewer Comments for Second Submission

**Comments Overview**

1. “Introduction. The authors are required to add more backgrounds about LA including the important papers suggested by the reviewer 1.”

- Thank you for this suggestion. The background has been updated to include more information about LA, including what is known for this compound (per Reviewer #2 request). Important papers suggested by Reviewer #1 have been added to the manuscript where applicable.

2. “Evaluation of the LA’s effects on TJ by claudin-4. Consider to evaluate the localization and amount of additional TJ transmembrane and/or plaque proteins, such as other claudins, occludin, JAMs, and ZO proteins. Alternatively, the authors should at least justify the use of claudin-4 by providing the reason why claudin-4 can represent the effect(s) on TJs with appropriate citations.”

- This is a valid point, and thank you for bringing this up. To be completely transparent, we did attempt probing (via western blotting) for other tight junction proteins, including occludin and claudin-2 using different antibodies for each. Unfortunately, these antibodies were not reactive in porcine tissue with our protocol.
- Additionally, although claudin-4 is not the only junctional protein that makes up the tight junction complex, we feel that this finding is significant enough to describe LA as having an effect on tight junction protein/structure given current information known about claudin-4 expression (highest in villous surface epithelium) while also leaving space to show that there may be other potential tight junction protein targets of LA. To better describe this, I have added a section to the Discussion that outlines other junctional components as well as recognizing the limitation of only showing data for one tight junction protein in this manuscript.
- I have also added references regarding the importance of claudin-4 in the tight junction structure and how its expression may be associated with restoration of barrier function after intestinal ischemia.

3. “LPS flux values: The reviewer 2 raises a concern that the value of LPS flux is too small. Please double-check the calculation. Also, consider to use Papp, which is easily compared with the results from other papers. Papp = (dQ/dt)/AxC0, where A = filter area, C0 = initial concentration in the donor compartment (See Van Itallie CM et al., J Cell Sci, 2007).”

- Thank you for bringing this point up. While we do agree the values appear small, we checked back to our previously collected data, and the data that is presented appears accurate to the best of our knowledge. We feel this data is still important and significant enough to include since we are comparing the flux values relative from one treatment to another, as on the graph. However, as the reviewer stated, it is important to recognize the small quantity of LPS that fluxed, and this can be added to the manuscript as a limitation/consideration.
- Additionally, we did attempt recalculating this data using the Papp equation suggested to us, and the coefficient values were still extremely small (x10^-6^). If you feel it necessary to represent the data as coefficient of permeability instead, we will happily change this part of the figure.

4. “As the reviewer 2 pointed out, the authors are recommended to improve the discussion of the results for the readers who are not familiar with the field.”

- Thank you and the reviewer for highlighting the potential lack of clarity of the Discussion. I have attempted to add to, clarify, and remove parts of the Discussion to enhance its clarity for those outside or new to the field. If you feel there are specific points that should be further clarified in the Discussion, I am more than happy to address these points.

Thank you again for all of your comments and thought,

- Zachary Slifer

Reviewer #1:

“In this submission to PLOS One, the authors present an experimental study to assess for the possibility of fragmentation of larazotide acetate (LA). The authors carry out western blot analysis of total protein isolated from uninjured and ischemia-injured porcine intestine, which showed aminopeptidase M enzyme presence in both tissue types, and mass spectrometry analysis of samples collected during ex vivo analysis confirmed formation of LA fragments. The authors conclude that LA stimulates repair of ischemic-injured epithelium at the level of the tight junctions, at an optimal dose of 1 μM LA. The authors find that higher doses were less effective because of inhibition by LA fragments.”

1. “I find this manuscript to be of interest to readers of PLOS One, and I am supportive of publication with a minor note/revision. In particular, there has been complementary studies using mass spectrometry to understand analysis of fragments, which should be mentioned:
   a. Journal of the American Society for Mass Spectrometry, 2014, 25, 1098-1113
   b. Am J Gastroenterol. 2012, 107, 1554–1562
   c. “In particular, these prior studies have used mass spectrometry techniques to probe fragments as well as LA molecules in these studies. With this minor note/modification, I would be supportive of publication in PLOS One.”

- Thank you for providing additional references to be included in this manuscript. The aforementioned references have been added as sources in appropriate locations, along with additional sources that help to further support information given regarding LA.

Thank you again for your support and comments of minor revisions,

- Zachary Slifer

Reviewer #2:

1. “The authors should make efforts to describe what is known for the LA compound. As mentioned in the manuscript, LA is in Phase III Clinical trials.”

- Thank you for suggesting this point. I absolutely agree that more information of LA should be included, and additional information about its properties, history, and clinical trials have been briefly included in the Introduction.

2. “Why the authors focus only on claudin-4? Tight junctions are complex with multiple types of claudins, occludin and junctional adhesion molecules. There is the matter of claudin shift, which indicates that a single claudin is not a good model.”

- This is a valid point and thank you for bringing this up. To be completely transparent, we did attempt probing (via western blotting) for other tight junction proteins, including occludin and claudin-2 using different antibodies for each. Unfortunately, these antibodies were not reactive in porcine tissue with our protocol. We initially did not state these negative findings in our manuscript, I have now included a statement about this negative data (as data not shown) to demonstrate to other readers that we did attempt to account for other junctional components.
- Additionally, although claudin-4 is not the only junctional protein that makes up the tight junction complex, we feel that this finding is significant enough to describe LA as having an effect on tight junction protein/structure while also leaving space to show that there may be other potential tight junction protein targets of LA To better describe this, I have added a section to the Discussion that outlines other junctional components as well as recognizing the limitation of only showing data for one tight junction protein in this manuscript.
- I have also added references regarding the importance of claudin-4 in the tight junction structure.

3. “pMLC is not well explained nor MLC or its phosphorylation.”

- Thank you for bringing my oversight to light. This section of the Discussion absolutely needed more information regarding the MLC pathway. Appropriate information with references has been added to the Discussion to link MLC to tight junction and intestinal permeability.

4. “LPS flux numbers are really small to be accurate measurements. “

- Thank you for bringing this point up. While we do agree the values appear small, we checked back to our previously collected data, and the data that is presented appears accurate to the best of our knowledge. We feel this data is still important and significant enough to include since we are comparing the flux values relative from one treatment to another, as on the graph. However, as you have highlighted, it is important to recognize the small quantity of LPS that fluxed, and this can be added to the manuscript as a limitation/consideration.
- Additionally, we did attempt recalculating this data using the Papp equation suggested to us, and the coefficient values were still extremely small (x10^-6^). If you or the editors feel it necessary to represent the data as coefficient of permeability instead, we will happily change this part of the figure.

5. “I can be more detailed in my review but essentially the discussion of the data was not clear for people that are outside the field nor for people new to the field.”

- Thank you for highlighting the potential lack of clarity of the Discussion. I have attempted to add to, clarify, and remove parts of the Discussion to enhance its clarity for those outside or new to the field. If you feel there are specific points that should be further clarified in the Discussion, I am more than happy to address these points.

Thank you again for all of your comments,

- Zachary Slifer
